# Effect of inspiratory muscle training on hypoxemia in obese patients undergoing painless gastroscopy: protocol for a single-center, double-blind, randomized controlled trial

**DOI:** 10.3389/fmed.2023.1269486

**Published:** 2023-09-14

**Authors:** Lijuan Yan, Xiao Wang, Kairong Du, Ying Liang

**Affiliations:** ^1^Department of Anesthesiology, The First Affiliated Hospital of Xiamen University, Xiamen, China; ^2^Department of Ultrasound, The First Affiliated Hospital of Xiamen University, Xiamen, China; ^3^Department of Pain Management, Zhongnan Hospital of Wuhan University, Wuhan, China; ^4^Department of Anesthesiology and Critical Care Medicine, Peking University First Hospital, Beijing, China

**Keywords:** inspiratory muscle training, obesity, hypoxemia, painless gastroscopy, randomized controlled trial, the Pittsburgh Sleep Quality Index

## Abstract

**Introduction:**

Obese patients who undergo painless gastroscopy are particularly prone to suffer from upper airway obstruction, respiratory depression, and subsequent hypoxemia. Despite adequate preoxygenation, the incidence of hypoxemia remains high. Recently, inspiratory muscle training (IMT) has been considered to be a promising strategy to increase respiratory muscle strength and endurance with the attendant improvement of pulmonary function. However, it remains unclear whether IMT is associated with a lower rate of hypoxemia in obese patients during this sedative procedure. This study aims to investigate the effectiveness of IMT used in obese patients who are scheduled for selective painless gastroscopy.

**Methods and analysis:**

This prospective, randomized controlled trial (RCT) will enroll 232 obese patients with a body mass index (BMI) of 35–39.9 kg·m^−2^ who undergo painless gastroscopy at the First Affiliated Hospital of Xiamen University. Subjects will be randomly assigned to two groups with a 1:1 ratio. Participants in both groups will receive IMT for 4 weeks prior to gastroscopy. The intervention group will receive IMT with a load of 30% of the maximal inspiratory pressure (Pi(max)) in the first week, with an increase of 10% per week since the following week, while the counterparts in the control group will not receive any load during the 4-week IMT. The primary outcome is the incidence of hypoxemia during painless gastroscopy. Secondary outcomes include the need for airway maneuvers, blood pressure changes, sleep quality assessment, pro-inflammatory cytokines levels, and monitoring of adverse events.

**Discussions:**

The outcomes of this study will offer invaluable guidance for the clinical implementation of IMT as a potential non-invasive preventive measure. Additionally, it stands to enrich our comprehension of anesthesia management and airway-related challenges in obese patients undergoing procedural sedation, which we anticipate will further contribute to addressing the turnaround concerns within high-volume, swiftly paced ambulatory endoscopy centers.

**Ethics and dissemination:**

This study has been approved by the Ethics Committee of the First Affiliated Hospital of Xiamen University (2022, No.091). The results will be submitted for publication in peer-reviewed journals.

**Trial registration number:**

China Clinical Trial Center (ChiCTR2200067041).

## Introduction

With the continuous improvement of living standards, an increasing number of patients are seeking safe and comfortable endoscopes to alleviate anxiety, pain, and discomfort during examinations. Hypoxemia is a common occurrence during upper gastrointestinal endoscopy, with an approximate incidence of 15% (1). It is important to note that more than one-third of the world’s population is overweight or obese patients (2), leading to a high incidence of hypoxemia, reaching 30.8%, during painless gastroscopy (3). Obesity is associated with a higher prevalence of hypoxemia due to factors such as mechanical compression of the chest wall, poor lung function, limited oxygen reserve capacity, increased oxygen consumption (4, 5), upper airway obstruction (6–8), and respiratory depression during procedural sedation (9). It is crucial to acknowledge that hypoxemia significantly contributes to various complications including brain damage, coronary ischemia, and tachycardia (9, 10). Therefore, optimal airway management to avoid hypoxemia is urgently needed for specific high-risk populations treated in high-volume, fast-paced, outpatient endoscopy centers.

Currently, there is no consensus on how to enhance oxygenation for obese patients during painless endoscopy procedures. The use of opioids and sedatives in such cases is closely linked to respiratory depression and airway blockage. However, recent evidence suggests that the combination of opioid-free analgesia esketamine and remimazolam can help mitigate sedation-induced respiratory depression and lower the occurrence of severe hypoxemia in obese patients undergoing painless gastrointestinal endoscopy (9). The existing strategies, including nasal cannula oxygenation supply, Wei nasal jet tubes, or supraglottic pulsatile jet oxygenation (8, 11), have provided partial prevention of airway collapse or obstruction (6). It is important to acknowledge that these approaches mainly target the upper airway rather than the smaller airways, leading to limited tolerance during the recovery phase and potentially unsuitability for high-risk hypoxemia patients. While the efficacy and safety of high-flow nasal cannula (HFNC) as a promising alternative for improving oxygenation in obese individuals have been established (2, 12–14), the practical implementation of HFNC in less developed regions remains unfeasible due to the requirement for specialized equipment, trained personnel, and the associated high costs (9). Therefore, it is crucial to identify an effective, practical, and non-invasive method to improve hypoxemia during painless gastroscopy for obese patients.

In recent times, the global impact of the coronavirus disease (COVID-19) has sparked considerable interest in inspiratory muscle training (IMT) as a fitness program, with the potential to alleviate symptoms of breathlessness, anxiety, and fatigue (15–17). IMT involves resistance-loaded muscle training using a small breathing device and is widely utilized in pulmonary rehabilitation settings (18). Its purpose is to enhance the performance of both primary and accessory respiratory muscles, improve physiological reserve, preserve the patency of smaller airways, increase maximal voluntary ventilation, and facilitate lung expansion (4, 15, 17, 19, 20). Several pieces of evidence have indicated that preoperative IMT can increase maximum inspiratory pressure (Pi(max)), decrease postoperative pulmonary complications, improve exercise endurance, and shorten hospital stays (21–23). Short-term IMT has shown significant improvements not only in oxygenation for patients undergoing lung cancer surgery (16) but also in reducing breathlessness and enhancing respiratory muscle function in individuals with long COVID-19 or obesity (24–26). In the intensive care unit, IMT has proven to be an effective method for reversing inspiratory muscle weakness, facilitating early weaning from mechanical ventilation, and enhancing weaning outcomes (27, 28) by increasing diaphragm thickness, an independent predictor of adverse outcomes measured by ultrasonography (29–31). Additionally, IMT has demonstrated positive effects on athletes, improving athletic performance and reducing inspiratory muscle fatigue (32). Presently, there are no reports of IMT being used to improve oxygenation during procedural sedation. However, we speculate that IMT could be a potential, practical, and non-invasive physical activity strategy to reduce the incidence of hypoxemia in obese patients undergoing procedural sedation.

To the best of our knowledge, this study represents the first attempt to assess the effect of IMT in reducing the incidence of hypoxemia during painless gastroscopy in obese individuals. For medical ethics and safety, this randomized controlled trial (RCT) will include 232 obese patients with a BMI ranging from 35 to 39.9 kg·m^−2^, all of whom are scheduled to undergo a 4-week IMT program prior to painless gastroscopy. Additionally, we aim to compare the impact of different IMT protocols on the requirement for airway maneuvers, blood pressure, sleep quality, pro-inflammatory cytokine levels, and any potential adverse events that may arise.

## Methods and analysis

### Study design

**Figure 1:**
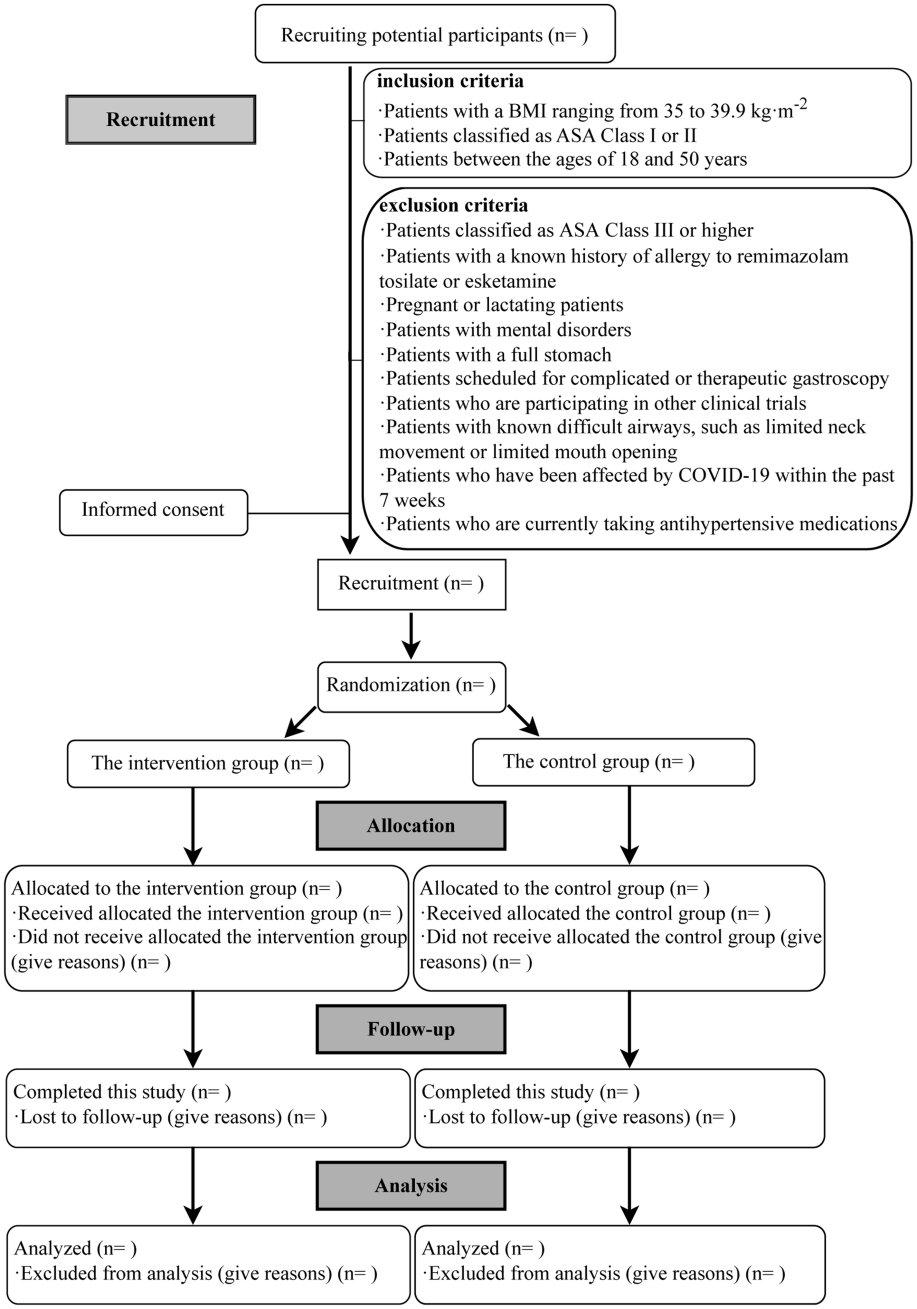
Flow chart of the study. BMI, body mass index; ASA, American Society of Anesthesiologists; COVID-19, the coronavirus disease.

This study is a single-center, double-blinded RCT scheduled to take place at the Endoscopy Center of the First Affiliated Hospital of Xiamen University. Participants will be assigned randomly to either the intervention or control group in a 1:1 ratio. The protocol adheres to the Standard Protocol Items: Recommendations for Interventional Trials guidelines (33) and will be conducted following the study’s flow chart (Figure 1).

To begin, participants will undergo IMT for 4 weeks before the gastroscopy procedure. In the intervention group, IMT will be administered with a load of 30% of the Pi(max) during the first week. Starting from the second week, the load will be increased by 10% each subsequent week. Conversely, the control group will not receive any load during the 4-week IMT period.

Subsequently, all patients will receive the same sedation strategy comprising remimazolam tosilate and esketamine. The gastroscopy procedure will be performed under a stable level of sedation. After the examination, patients will be transferred to the post-anesthesia care unit (PACU) and discharged once their modified Aldrete score reaches 9.

Lastly, all data will be collected as per the schedule outlined in Table 1.

**Table 1:** The schedule of enrollment, interventions, and assessments.

Study period	Screening	Allocation	IMT		Anesthesia and examination		PACU	Follow-up		
		T1		T2		T3		T4	T5	T6
**Enrollment**										
Inclusion criteria	X									
Exclusion criteria	X									
Informed consent	X									
Randomization	X									
Demographics data	X									
Physical examination data	X									
Allergy history	X									
Comorbidities	X									
**Interventions**										
The intervention group			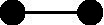							
The control group			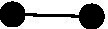							
**Assessments**										
**Clinical monitoring**										
(1) Vital signs	X	X		X	X		X			
(2) Pi(max)		X								
(3) MOAA/S				X	X					
(4) BIS				X	X					
**Outcome assessment**										
(1) The incidence of hypoxemia					X					
(2) The need for airway maneuvers					X					
(3) Blood pressure		X		X	X		X			
(4) Sleep quality		X		X				X	X	X
(5) Pro-inflammatory cytokines levels		X		X		X				
(6) Adverse events					X		X			
**Requirement for vasoactive agents**					X		X			
**Need for rescue sedatives**					X		X			

IMT, inspiratory muscle training; PACU, post-anesthesia care unit; Pi(max), maximum inspiratory pressure; MOAA/S, Modified Observer’s Assessment of Alertness and Sedation; BIS, bispectral index; T1, 1 day before IMT; T2, immediately prior to anesthesia; T3, at the conclusion of the gastroscopy; T4, on the night of the gastroscopy; T5, 1 week after the gastroscopy; T6, 4 weeks after the gastroscopy.

### Participant enrollment

Between September 2023 and August 2024, potential subjects for this study will be recruited from the outpatient clinic of the Department of Anesthesiology. This clinic handles a monthly volume of over 2000 patients undergoing painless gastroscopy, and the prevalence of obesity in this population exceeds 1 percent. Out of the eligible candidates, a total of 232 patients will be enrolled. Before enrollment, these individuals will receive comprehensive information regarding the study’s purpose, procedures, considerations, potential benefits and risks, as well as any associated costs. They will be fully informed and have the opportunity to provide voluntary consent to participate. Both the subjects and their authorized representatives will be responsible for maintaining the confidentiality of the received protocol. Written informed consent will be obtained from all subjects prior to their enrollment. Participants will have the freedom to withdraw from the study at any point without incurring any liability, and such withdrawal will not impact their subsequent gastroscopy procedures.

### Participants’ inclusion and exclusion criteria

All potential subjects will undergo a screening process based on the defined inclusion and exclusion criteria. If a subject meets the following shedding criteria, they will be excluded from this study.

### Inclusion criteria

1. Patients with a body mass index (BMI) ranging from 35 to 39.9 kg·m^−2^.
2. Patients classified as American Society of Anesthesiologists (ASA) Class I or II.
3. Patients between the ages of 18 and 50 years.

### Exclusion criteria

1. Patients classified as ASA Class III or higher.
2. Patients with a known history of allergy to remimazolam tosilate or esketamine.
3. Pregnant or lactating patients.
4. Patients with mental disorders.
5. Patients with a full stomach.
6. Patients scheduled for complicated or therapeutic gastroscopy.
7. Patients who are participating in other clinical trials.
8. Patients with known difficult airways, such as limited neck movement or limited mouth opening.
9. Patients who have been affected by COVID-19 within the past 7 weeks.
10. Patients who are currently taking antihypertensive medications.

### Shedding criteria

1. Patients or their authorized representatives request to withdraw at any stage of the study.
2. Severe complications arise during the examination, necessitating prolonged examination or emergency surgery.
3. Severe hypoxemia occurs during the study, requiring endotracheal intubation.

### Intervention

This study aims to assess the impact of IMT on enhancing oxygenation levels during painless gastroscopy in individuals with a BMI of 35–39.9 kg·m^−2^. Upon identifying the participants, an intervention delivery schedule will be established. Before undergoing gastroscopy, participants will follow a 4-week IMT intervention protocol based on their assigned group. The timeline for enrollment, interventions, and assessments is outlined in detail in Table 1. All data will be collected according to the participant’s specific timeline, which includes the following time points: T1: 1 day before IMT, T2: immediately prior to anesthesia, T3: at the conclusion of the gastroscopy, T4: on the night of the gastroscopy, T5: 1 week after the gastroscopy, and T6: 4 weeks after the gastroscopy.

For participants assigned to the intervention group, a research assistant will measure their Pi(max) using the IMT device (POWER^®^breathe, Tianrui, Jiangsu). During the measurement process, patients will be seated and wear a nasal clip. They will exhale slowly and completely, followed by inhaling with maximum force and holding it for at least 1.5 s. The inspiratory pressure at this point will be recorded. This process will be repeated three times, and the average of the recorded inspiratory pressures will be considered as the Pi(max).

POWER^®^breathe follows a standard practice of conducting 30 inspiratory breaths twice a day (20). The intervention group will undergo IMT using a load equivalent to 30% of their Pi(max) during the first week. Starting the following week, the load will be increased by 10% each week for a total of three adjustments. On the other hand, participants in the control group will not receive any load during the 4-week IMT. It is important to note that participants will not be informed about their assignment to either the intervention or control group.

### Monitoring and standard practice-based anesthesia protocol

To enhance subject compliance with IMT, all participants will receive comprehensive information about the procedure and specifics of IMT. Additionally, they will be remotely instructed in real-time by an independent research assistant using video software. In this study, adjusted body weight will be utilized, calculated as follows: ideal body weight + 0.4 × (total body weight-ideal body weight) (9). In order to mitigate the risk of potential complications during sedation, we will ensure the presence of standby equipment, including HFNC, anesthesia machines, laryngeal masks, endotracheal catheters, and laryngoscopes.

Upon arrival at the endoscopy center, all subjects will be positioned in the left decubitus position and undergo routine monitoring, which includes non-invasive blood pressure, electrocardiography, heart rate (HR), peripheral oxygen saturation (SpO_2_), respiratory rate, and end-tidal carbon dioxide levels.

The procedure will be conducted in the following steps:

Firstly, before administering procedural sedation, patients will receive oxygen through a nasal tube at a flow rate of 3 L∙min^−1^ and will be instructed to take 10 deep breaths.

Secondly, intravenous administration of esketamine at a dose of 0.5 mg·kg^−1^ will be carried out within 10 s. Simultaneously, remimazolam tosilate will be infused using a micromedicine infusion pump at a rate of 12 mg·kg^−1^·h^−1^ until the Modified Observer’s Assessment of Alertness and Sedation (MOAA/S) score reaches 1, as indicated in Table 2.

**Table 2:** Modified observer’s assessment of alertness/sedation scale.

Score	Responsiveness
5	Responds readily to name spoken in normal tone
4	Lethargic response to name spoken in normal tone
3	Responds only after name is called loudly and/ or repeatedly
2	Responds only after mild prodding or shaking
1	Responds only after painful trapezius squeeze
0	Does not respond to painful trapezius squeeze

Thirdly, to prevent the tongue from falling back, a suitable nasopharyngeal airway with end-tidal carbon dioxide monitoring will be carefully inserted into the patient’s nasal cavity. At this stage, gastroscopy will be performed by an experienced endoscopist.

Throughout the procedure, remimazolam tosilate dosage will be dynamically adjusted based on the reference bispectral index (BIS), which is determined when the MOAA/S score reaches 1, to maintain a stable level of sedation.

If any involuntary movements, such as coughing, raising the head, or body shifting, are observed in a patient, an immediate infusion of remimazolam tosilate at a dose of 0.05 mg·kg^−1^ will be administered. If such movements persist after 1 min, a reapplication of remimazolam tosilate may be necessary. After the examination, all patients will be transferred to the PACU for recovery.

In cases where the heart rate is less than 45 beats per minute or exceeds 100 beats per minute, Atropine at a dose of 0.5 mg or Esmolol at a dose of 1 mg·kg^−1^ will be administered separately to address the situation. Similarly, if the mean arterial pressure deviates by more than 20% from the baseline, Phenylephrine at a dose of 40 μg or Urapidil hydrochloride at a dose of 12 mg will be promptly administered accordingly.

If a patient’s SpO_2_ falls below 90%, the experienced anesthesiologist must differentiate between respiratory obstruction and respiratory depression (34). For respiratory obstruction, the anesthesiologist will apply jaw-lift. On the other hand, respiratory depression will be managed using appropriate measures such as positive-pressure ventilation using a bag-mask.

If deemed necessary, flumazenil will be employed to counteract the effects of remimazolam tosilate. However, in the event of severe hypoxemia and if the aforementioned methods prove ineffective, the anesthesiologist will promptly administer either a laryngeal mask or perform endotracheal intubation to ensure adequate airway management.

### Primary outcomes

The primary outcome of this study is the incidence of hypoxemia during painless gastroscopy. Hypoxemia will be defined as a SpO_2_ level below 90%.

### Secondary outcomes

The secondary outcomes encompass several measures, including the need for airway maneuvers, blood pressure changes, sleep quality assessment, pro-inflammatory cytokine levels, and monitoring of adverse events such as bradycardia, tachycardia, nausea, and vomiting.

For this study, airway maneuvers will be defined as jaw-lift, positive-pressure ventilation with a bag-mask, insertion of a laryngeal mask, or endotracheal intubation.

Blood pressure will be measured at different time points: before IMT, prior to anesthesia, during the examination, and in the PACU.

Sleep quality and duration will be assessed using the Pittsburgh Sleep Quality Index (PSQI), a 19-item questionnaire (Table 3), at T1, T2, T4, T5, and T6. A patient scoring greater than 5 on the PSQI will be classified as a poor sleeper (35).

**Table 3:** The questionnaire of Pittsburgh Sleep Quality Index.

1. During the past month, when have you usually gone to bed?				
2. During the past month, how long (in minutes) has it taken you to fall asleep each night?	≤15 min (0)	16 ~ 30 min (1)	31 ~ 60 min (2)	>60 min (3)
3. During the past month, when have you usually gotten up in the morning?				
4. During the past month, how many hours of actual sleep did you get that night? (This may be different than the number of hours you spend in bed)	>7 h (0)	6 ~ 7 h (1)	5 ~ 6 h (2)	<5 h (3)
5. During the past month, how often have you had trouble sleeping because you…	Not during the past month (0)	Less than once a week (1)	Once or twice a week (2)	Three or more times a week (3)
a. Cannot get to sleep within 30 min	(0)	(1)	(2)	(3)
b. Wake up in the middle of the night or early morning	(0)	(1)	(2)	(3)
c. Have to get up to use the bathroom	(0)	(1)	(2)	(3)
d. Cannot breathe comfortably	(0)	(1)	(2)	(3)
e. Cough or snore loudly	(0)	(1)	(2)	(3)
f. Feel too cold	(0)	(1)	(2)	(3)
g. Feel too hot	(0)	(1)	(2)	(3)
h. Have bad dreams	(0)	(1)	(2)	(3)
i. Have pain	(0)	(1)	(2)	(3)
j. Other reason(s), please describe, including how often you have had trouble sleeping because of this reason(s)	(0)	(1)	(2)	(3)
6. During the past month, how often have you taken medicine (prescribed or “over the counter”) to help you sleep?	Not during the past month (0)	Less than once a week (1)	Once or twice a week (2)	Three or more times a week (3)
7. During the past month, how often have you had trouble staying awake while driving, eating meals, or engaging in social activity?	Not during the past month (0)	Less than once a week (1)	Once or twice a week (2)	Three or more times a week (3)
8. During the past month, how much of a problem has it been for you to keep up enthusiasm to get things done?	Not during the past month (0)	Less than once a week (1)	Once or twice a week (2)	Three or more times a week (3)
9. During the past month, how would you rate your sleep quality overall?	Very good (0)	Fairly good (1)	Fairly bad (2)	Very bad (3)

Component		Componen*t* score
Sleep quality	#9 Score	
Time to fall asleep	#2 Score + #5a Score (0 = 0; 1 ~ 2 = 1; 3 ~ 4 = 2; 5 ~ 6 = 3)	
Bed time	#4 Score	
Sleep efficiency	#4/(#3 – #1) * 100% (>85% = 0, 75–84% = 1, 65–74% = 2, <65% = 3)	
Somnipathy	# sum of scores 5b to 5j (0 = 0; 1 ~ 9 = 1; 10 ~ 18 = 2; 19 ~ 27 = 3)	
Hypnotics	#6 Score	
Daytime dysfunction	#7 score + #8 score (0 = 0; 1 ~ 2 = 1; 3 ~ 4 = 2; 5 ~ 6 = 3)	
**Score of Pittsburgh Sleep Quality Index**		

This scale is designed to record your own assessment of any sleep difficulty you might have experienced. Please complete this scale at T1, T2, T4, T5, and T6. All scales will be finally e-mailed to designated anesthesiologist. (T1: one day before inspiratory muscle training, T2: the moment before anesthesia, T4: the night of gastroscopy, T5: 1 week after gastroscopy, T6: 4 weeks after gastroscopy).

At three different time points, namely T1, T2, and T3, 5 mL of venous blood will be collected from the patient’s elbow. The collected blood samples will be processed through conventional centrifugation to separate the plasma. Subsequently, radioimmunoassay will be employed to measure the plasma levels of pro-inflammatory cytokines, including tumor necrosis factor-alpha (TNF-α) and interleukin-6 (IL-6).

### Data collection, handling, and monitoring

We will gather the following data for this study:

1. Demographic data, including age, gender, and ASA physical status classifications.
2. Physical examination data, including HR, mean arterial pressure (MAP), SpO_2_, respiratory rate, end-tidal carbon dioxide levels, Pi(max), BMI, neck circumference, and waist-hip ratio.
3. Data related to the primary and secondary outcomes as mentioned earlier.
4. Requirement for vasoactive agents, such as atropine, esmolol, phenylephrine, and urapidil hydrochloride.
5. Need for rescue sedatives.

In order to prevent any loss of follow-up, a research assistant who knows the group assignment will reach out to patients from time to time. Their purpose is to remind patients to fill out the PSQI sheets.

To ensure data integrity and security, an independent monitor will oversee the data collection process. Data collectors will enter all raw data into Excel spreadsheets, which will then be stored in a separate database with restricted access using passwords. Additionally, original data and images will be securely stored on the research center’s computer, accessible only with login passwords. Unauthorized access to the database or computer will be strictly prohibited without the monitor’s permission.

In case of any adverse events, immediate attention will be given, followed by detailed recording and continuous follow-up. The Ethics Committee will periodically review the project’s progress and decide whether it should be continued.

### Randomization

All enrolled patients will be randomly assigned to either the intervention or control groups in a 1:1 ratio. The random number sequence will be generated using the Statistical Product and Service Solutions (IBM SPSS, V.20.0) statistical software. Grouping information will be securely stored in opaque envelopes.

### Blinding

The independent research assistant will have the responsibility of explaining the procedure and details of IMT and measuring the Pi(max) for all participants. The research assistant will conduct remote instruction and real-time monitoring of the participants through video software, making them unblinded to the process. However, both the participants and the research assistant will be explicitly instructed not to disclose any details to other researchers involved in the study.

On the other hand, the anesthesiologist, the endoscopist, data collectors, laboratory technicians, and biostatistician will be blinded to the intervention protocols and group allocation to ensure unbiased evaluation.

### Sample size

We based our calculations on an incidence of the primary endpoint at 40% in the control group and 20% in the intervention group. As a result, we arrived at a revised sample size of 105 participants for each group to yield 90% power with a type I error of 0.05. Anticipating a potential dropout rate of 10%, our target enrollment is set at 232 participants in total, evenly distributed with 116 participants per group for the study.

### Statistical analysis

An independent biostatistician, not involved in our trial, will analyze all the data using optimal software. We will use the Last Observation Carried Forward (LOCF) imputation method to handle missing data. However, this study will not include interim analyses.

Continuous variables will be assessed using either the *t*-test or Wilcoxon rank test, depending on the nature of the data. For the analysis of counting data, the chi-square test or Fisher’s exact test will be employed for comparison. Statistical significance will be defined as *p*<0.05.

## Discussion

Hypoxemia is a significant factor contributing to a high incidence of complications and mortality (9, 10). Finding an appropriate precautionary measure to prevent hypoxemia during procedural sedation, particularly in obese patients, is both necessary and intriguing. This study represents the first RCT to assess the impact of IMT on hypoxemia in obese individuals undergoing painless gastroscopy. The results of this study will provide valuable guidance for using IMT as a precautionary measure in clinical settings. It will also enhance our understanding of anesthesia management and airway-related concerns during procedural sedation for obese patients. Furthermore, the study will help shorten turnaround times in busy ambulatory endoscopy centers.

### Strengths of the study

First, our study design focuses on IMT as a practical, minimally invasive, well-tolerated, non-pharmacological physical activity strategy (25). The IMT device is simple to use, and compact in size, allowing for easy reproducibility and potential widespread application in clinical settings.

Second, the infusion dose will be precisely adjusted based on the individualized reference BIS value when the MOAA/S reaches 1, ensuring a consistent and stable level of sedation. Recent evidence has demonstrated that BIS-based titration of hypnotics can result in stable levels of sedation (36, 37). Previous studies have calculated the sedative dose based on adjusted body weight or ideal body weight, which may not accurately reflect the actual sedative dose needed. However, in obese patients, both fat and muscle mass are increased, and their proportions differ.

Third, in this study, the sedation strategy consists of remimazolam tosilate and esketamine, both of which have shown beneficial effects in reducing severe hypoxemia in obese individuals undergoing painless gastrointestinal endoscopy (9). In China, a common sedation strategy for procedural sedation involves propofol combined with low-dose opioids. However, propofol is associated with a high risk of cardiopulmonary depression events, which may be further exacerbated in obesity due to their heightened respiratory vulnerability and increased sensitivity to propofol (38). In contrast, remimazolam is a new benzodiazepine drug with rapid onset and recovery and less cardiorespiratory depression. Opioids can induce and worsen sleep-disordered breathing, especially in patients with morbid obesity (9). Ketamine, proposed for use in opioid-free anesthesia (39), does not significantly affect respiration. Esketamine, the S-enantiomer of ketamine, has a higher clearance rate.

Fourth, our study’s findings may hold promising implications for clinical application. Existing evidence has highlighted the potential of short-term loaded IMT to enhance respiratory muscle function, subsequently improving oxygenation levels (24–26). Should this intervention effectively mitigate the occurrence of hypoxemia among obese patients during painless gastroscopy, it will result in decreased reliance on airway interventions and fewer sedation-related complications. Consequently, this may streamline procedural sedation, delivering notable advantages to busy ambulatory endoscopy centers. Furthermore, these favorable outcomes will promote the progress and adoption of comfort-focused medical practices, especially within these distinct high-risk populations.

Last, we will explore its impact on blood pressure, sleep quality, and pro-inflammatory cytokines in obese individuals undergoing painless gastroscopy. Both procedural sedation and obesity can have negative effects on sleep and the cardiopulmonary system (17, 40), and pro-inflammatory cytokines play a role in sleep regulation and are associated with various diseases (41). Several studies have indicated that IMT can be used as an auxiliary means to reduce the tendency of upper airway collapse during sleep and improve sleep quality (17).

### Limitations

This study has several limitations that merit acknowledgment. Firstly, to mitigate potential bias arising from a wide BMI range, we will intentionally focuse solely on obese individuals with a BMI ranging from 35 to 39.9 kg·m^−2^ as our study subjects. Notably, individuals classified as morbidly obese, who may necessitate more intricate and cautious airway management during procedural sedation, will not be included. This approach may potentially impact the observed outcomes. As a result, patients with a BMI exceeding 40 kg·m^−2^ will not be part of the current study. Nevertheless, we remain committed to continually accumulating data on morbidly obese individuals in forthcoming experiments, intending to design a more tailored study scheme for this particular patient group. Additionally, it’s important to acknowledge that this study is essentially a single-center pilot study. Despite its notable reliability and validity, the sample size remains limited. Following completion, all insights and data will undergo thorough reanalysis to refine the design and execution of subsequent larger-scale studies.

### Ethics and dissemination

This RCT was designed in adherence to the principles outlined in the Declaration of Helsinki. The trial received ethical approval from the Ethics Committee of the First Affiliated Hospital of Xiamen University on December 28, 2022 (Scientific Research Ethics Review 2022, No.091). Subsequently, the protocol amendment was thoroughly discussed and unanimously confirmed by all investigators on March 7, 2023. To ensure transparency and accessibility, this trial has been registered with the China Clinical Trial Center (ChiCTR2200067041) since December 26, 2022.

Prior to their participation, all prospective participants will be fully informed about the procedure and potential risks associated with this study. They will be requested to provide informed consent, demonstrating their voluntary agreement to take part in the research.

Upon completion, all research findings will be disseminated through publication in peer-reviewed journals in the form of papers. Furthermore, to promote open access and facilitate public awareness, the full protocol and research data will be made available for public access at the appropriate time.

## Ethics statement

The studies involving humans were approved by the Ethics Committee of the First Affiliated Hospital of Xiamen University. The studies were conducted in accordance with the local legislation and institutional requirements. The participants provided their written informed consent to participate in this study. Written informed consent was obtained from the individual(s) for the publication of any potentially identifiable images or data included in this article.

## Author contributions

LY: Conceptualization, Formal Analysis, Funding acquisition, Investigation, Methodology, Project administration, Resources, Software, Supervision, Validation, Visualization, Writing – original draft. XW: Conceptualization, Formal Analysis, Investigation, Methodology, Project administration, Resources, Software, Visualization, Writing – original draft. KD: Conceptualization, Data curation, Formal Analysis, Investigation, Methodology, Resources, Software, Visualization, Writing – review & editing, Validation. YL: Conceptualization, Data curation, Formal Analysis, Investigation, Methodology, Resources, Software, Visualization, Writing – review & editing, Validation.

## Funding

The author(s) declare financial support was received for the research, authorship, and/or publication of this article. This work was supported by Natural Science Foundation of Xiamen, China (grant number: 3502Z20227345) and Clinical Special Project of Fujian University of Traditional Chinese Medicine (grant number: XB2022184).

## Conflict of interest

The authors declare that the research was conducted in the absence of any commercial or financial relationships that could be construed as a potential conflict of interest.

## Publisher’s note

All claims expressed in this article are solely those of the authors and do not necessarily represent those of their affiliated organizations, or those of the publisher, the editors and the reviewers. Any product that may be evaluated in this article, or claim that may be made by its manufacturer, is not guaranteed or endorsed by the publisher.
