# Supplementary material for: Effect of inspiratory muscle training on hypoxemia in obese patients undergoing painless gastroscopy: protocol for a single-center, double-blind, randomized controlled trial
Source: Front Med (Lausanne). 2023 Sep 14;10:1269486. doi: 10.3389/fmed.2023.1269486 (PMC10542889; doi:10.3389/fmed.2023.1269486)
Supplement: Supplementary file 2 [file Presentation_2.PDF]

知情同意书·知情告知页

Informed Consent Form - Information Page

方案名称：吸气肌训练改善中度肥胖患者无痛胃镜检查中氧合状态的临床有效性研究

Clinical Efficacy Study of Inspiratory Muscle Training to Improve Oxygenation Status in Moderately Obese Patients Undergoing Painless Gastroscopy

方案版本号及版本日期：V3.0　2023.02.26
Protocol Version and Date: V3.0 2023.02.26

知情同意书版本号及版本日期：V3.0　2023.02.26
Informed Consent Form Version and Date: V3.0 2023.02.26

主要研究者：闫丽娟
Principal Investigator: Lijuan Yan

申办者：厦门大学附属第一医院麻醉科
Sponsor: Department of Anesthesiology, The First Affiliated Hospital of Xiamen University, Xiamen, Fujian, China

尊敬的受试者：

您被邀请参加 吸气肌训练改善中度肥胖患者无痛胃镜检查中氧合状态的临床有效性研究。请仔细阅读本知情同意书并慎重做出是否参加本项研究的决定。当您的研究医生或者研究人员和您讨论知情同意书的时候，您可以让他/她给您解释您看不明白的地方。我们鼓励您在做出参与此项研究的决定之前，和您的家人及朋友进行充分讨论。若您正在参加别的研究，请告知您的研究医生或者研究人员。本研究的目的、背景、研究过程及其他　重要信息如下：

Dear Participant,

You are invited to participate in a clinical efficacy study titled "Inspiratory Muscle Training to Improve Oxygenation Status in Moderately Obese Patients Undergoing Painless Gastroscopy." Please carefully read this informed consent form and consider your decision to participate in this study. If there are any parts that you do not understand, you may ask your study doctor or research personnel to explain them to you. We encourage you to discuss this study thoroughly with your family and friends before deciding to participate. If you are currently participating in any other study, please inform your study doctor or research personnel. The purpose, background, research process, and other important information about this study are as

follows:

一、 研究背景

Section One: Study Background

胃镜检查是诊断和治疗上消化疾病的常用手段。在静脉麻醉或镇静的胃镜检查期间，由短暂性呼吸抑制和气道阻塞引起的低氧血症很常见。然而，对于肥胖患者，肥胖因素常累及呼吸系统，发生气道解剖结构改变、潮气量和功能残气量下降、胸壁和肺的顺应性降低，弹性及肌肉工作负荷增加，呼吸肌力量和耐力下降，氧耗水平增加及疲劳感加重，镇静麻醉过程中更容易出现低氧血症，对麻醉医生的麻醉管理提出很大的挑战。

Gastroscopy is a common method for diagnosing and treating upper digestive tract disorders. During gastroscopy under intravenous anesthesia or sedation, hypoxemia caused by transient respiratory suppression and airway obstruction is frequently observed. However, for obese patients, obesity-related factors often affect the respiratory system, leading to changes in airway anatomy, reduced tidal volume and functional residual capacity, decreased compliance of the chest wall and lungs, increased elasticity and muscular workload, diminished respiratory muscle strength and endurance, elevated oxygen consumption, and heightened fatigue sensation. As a result, obese patients are more susceptible to hypoxemia during the sedation and anesthesia process, posing significant challenges to anesthesiologists in anesthesia management.

先前的研究表明，短期（2~4 周）吸气肌训练（inspiratory muscle training, IMT）可改善肥胖患者的肺功能、有氧运动能力、呼吸肌力量和耐力、四肢肌肉血管扩张、心血管控制和生活质量的改善。长期（12 周）IMT 可提高病态肥胖患者的最大吸气压和最大通气量，故术前 IMT 可能是一项预防肥胖患者术后肺部并发症的预防手段。

Previous studies have indicated that short-term (2-4 weeks) Inspiratory Muscle Training (IMT) can improve lung function, aerobic capacity, respiratory muscle strength and endurance, peripheral muscle vasodilation, cardiovascular control, and quality of life in obese patients. Long-term (12 weeks) IMT can enhance maximal inspiratory pressure and maximal ventilation in morbidly obese patients. Therefore, preoperative IMT may serve as a preventive measure against postoperative pulmonary complications in obese patients.

据我们所知，肥胖患者无痛胃镜检查期间容易发生低氧血症并无有效的干预措施，对于病态肥胖患者将面临麻醉医生拒绝为其镇静麻醉的可能。目前，并无肥胖患者无痛胃镜检查前进行 IMT 的研究报道。本研究旨在探究短期 IMT 改善肥胖患者无痛胃镜检查期间低氧血症的有效性。

To the best of our knowledge, there are no effective interventions to prevent hypoxemia in obese patients during painless gastroscopy, and severely obese patients may face the possibility of anesthesia refusal for sedation. Currently, there are no studies reporting the use of Inspiratory Muscle Training (IMT) prior to painless

gastroscopy in obese patients. This study aims to investigate the effectiveness of short-term IMT in improving hypoxemia during painless gastroscopy in obese patients.

二、 研究目的
Section Two: Study Objectives

本研究的研究目的是探究短期 IMT 改善中度肥胖患者无痛胃镜检查期间低氧血症的有效性。

The purpose of this study is to investigate the effectiveness of short-term Inspiratory Muscle Training (IMT) in improving hypoxemia during painless gastroscopy in moderately obese patients.

三、研究过程
Section Three: Study Procedures

1. 多少人将参与这项研究？
1. How many people will take part in this study?

大约 56 人将参与在厦门大学附属第一医院开展的本项研究。

Approximately 56 individuals will participate in this study conducted at the First Affiliated Hospital of Xiamen University.

2. 研究步骤
2. Study Procedures

如果您同意参加本研究，请您签署这份知情同意书。确定您可以参加本研究后，您将在胃镜检查前 4 周被随机分组至干预组或对照组，无论您在哪一组，均需进行 4 周的 IMT 后再行无痛胃镜检查。胃镜检查当天，所有患者先静脉缓慢（10s）注射艾司氯胺酮 0.5 $mg \cdot kg^{-1}$，同时，静脉泵注瑞马唑仑 12 $mg \cdot kg^{-1} \cdot h^{-1}$，直至改良警觉/镇静量表（MOAA/S）评分＝1 时，开始胃镜检查。如果患者检查期间出现体动，可追加瑞马唑仑 0.05 $mg \cdot kg^{-1}$，记录两组患者 HR、MAP、S_PO_2、RR、PSQI、TNF-α 和 IL-6 及不良事件。

If you agree to participate in this study, please sign this informed consent form. Once your eligibility for the study is confirmed, you will be randomly assigned to either the intervention group or the control group four weeks before the gastroscopy. Regardless of the group, all participants will undergo four weeks of Inspiratory Muscle Training (IMT) before undergoing the painless gastroscopy. On the day of the gastroscopy, intravenous administration of esketamine at a dose of 0.5 $mg \cdot kg^{-1}$ will be carried out within 10 seconds. Simultaneously, remimazolam tosilate will be infused using a micromedicine infusion pump at a rate of 12 $mg \cdot kg^{-1} \cdot h^{-1}$ until the Modified Observer’s Assessment of Alertness and Sedation (MOAA/S) score reaches 1. The gastroscopy will commence at this time. In case of patient movement during the procedure, additional remimazolam tosilate (0.05 $mg \cdot kg^{-1}$) can be administered. Heart

rate (HR), mean arterial pressure (MAP), oxygen saturation (SpO_2), respiratory rate (RR), Pittsburgh Sleep Quality Index (PSQI), TNF-α, IL-6, and adverse events will be recorded for both groups.

3. 这项研究会持续多久？

3. How long will this study last?

本研究自麻醉镇静至患者离开内镜中心。

您可以在任何时间选择退出研究而不受到任何惩罚，也不会丧失您本应获得的任何利益。然而，如果在研究途中您决定退出本研究，我们鼓励您先和您的医生商议。考虑到您的安全性问题，有可能在退出后，会进行一次相关检查。

This study will continue from the anesthesia sedation until the patient departs from the endoscopy center.

You have the right to withdraw from the study at any time without any penalties or loss of benefits you would normally receive. However, if you decide to withdraw during the course of the study, we encourage you to consult with your doctor first. For safety considerations, a follow-up examination may be conducted after your withdrawal.

四、风险与受益

Section Four: Risks and Benefits

1. 参加本研究的风险是什么？

1. What are the risks of participating in this study?

本研究采用吸气肌训练的干预方法，是康复医学中的常用呼吸功能锻炼的方法之一，无任何额外风险。

This study employs the intervention of Inspiratory Muscle Training, which is a common respiratory exercise method used in rehabilitative medicine. There are no additional risks associated with this intervention.

2. 参加研究有什么受益？

2. What are the benefits of participating in the study?

我们希望从您参与的本研究中得到的信息，将有助于医师临床工作的有效开展，在将来能够使与您病情相同的病人获益。

We hope that the information obtained from your participation in this study will contribute to the effective clinical practice of physicians and potentially benefit patients with similar conditions in the future.

五、研究结果的使用和个人信息的保密

Section Five: Use of Study Results and Confidentiality of Personal Information

在您和其他受试者的理解和协助下，通过本项目研究的结果可能会在医学杂

志上发表，但是我们会按照法律的要求为您的研究记录保密。研究受试者的个人信息将受到严格保密，除非应相关法律要求，您个人信息不会被泄露。必要时，政府管理部门和医院伦理委员会及其它相关研究人员可以按规定查阅您的资料。

With your understanding and cooperation, the results of this research project may be published in medical journals. However, we will ensure the confidentiality of your research records as required by law. The personal information of research participants will be kept strictly confidential, and your personal information will not be disclosed unless required by relevant laws. When necessary, government regulatory agencies, hospital ethics committees, and other relevant researchers may access your information as stipulated.

六、关于研究费用及相关补偿

Section Six: Research Costs and Compensation

1. 研究所用的药物及相关检查费用

1. Costs of Medication and Related Examinations

治疗方案中，受试者只需支付正常术前检查检验和手术相关费用。本研究中调查问卷和研究者观察的形式，不会产生任何试验相关的额外费用。TNF-α 和 IL-6 的实验室费用由研究者支付。

Under the treatment plan, participants will only need to cover the regular preoperative tests, examinations, and procedure-related expenses. The forms of questionnaire surveys and researcher observations in this study will not incur any additional trial-related charges. Laboratory expenses for TNF-α and IL-6 will be covered by the researchers.

2. 参加研究的补偿

2. Compensation for Participation in the Study

鉴于本研究经费有限，参加本研究无任何酬劳，敬请理解。

随访的方式为研究者与被检查者当面交流，不会让您产生任何交通费用。

Given the limited funding for this study, kindly understand that there will be no remuneration for participating in this study.

Face-to-face communication between researchers and participants for follow-up purposes will not incur any transportation expenses.

3. 发生损伤后的补偿

3. Compensation for Injuries

如果您的健康因参加这项研究而发生与研究相关的损害，请立即通知研究医生，他们将负责对您采取适当的治疗措施。厦门大学附属第一医院将承担治疗费用及按照中国有关规定对您给予相应的经济补偿。若因发生与研究相关的死亡，您的受赡养人将得到相应的经济补偿。厦门大学附属第一医院提供法律上与经济上的担保，但因未遵循研究方案而导致的损伤，厦门大学附属第一医院不予补偿。

If your health is adversely affected due to your participation in this study, please

inform the research doctor immediately. They will be responsible for providing appropriate medical treatment. The First Affiliated Hospital of Xiamen University will cover the treatment expenses and provide the necessary economic compensation according to relevant regulations in China. In the unfortunate event of a study-related death, your dependents will receive appropriate financial compensation. The First Affiliated Hospital of Xiamen University offers legal and financial guarantees, but injuries resulting from failure to adhere to the study protocol will not be compensated.

七、受试者的权利和责任
Section Seven: Participant's Rights and Responsibilities

1. 您的权利
1. Your Rights

在参加研究的整个过程中，您都是自愿的。如果您决定不参加本研究，也不会影响您应该得到的其他治疗。如果您决定参加，会要求您在这份书面知情同意书上签字。您有权在试验的任何阶段随时退出试验而不会遭到歧视或受到不公平的待遇，您相应医疗待遇与权益不受影响。

Throughout your participation in this study, your involvement is entirely voluntary. If you decide not to participate in this study, it will not affect other treatments you should receive. If you choose to participate, you will be asked to sign this written informed consent form. You have the right to withdraw from the trial at any stage without discrimination or unfair treatment. Your corresponding medical care and rights will not be affected.

2. 您的责任
2. Your Responsibilities

作为受试者，您需要提供有关自身病史和当前身体状况的真实情况；告诉研究医生自己在本次研究期间所发现的任何不适；不得服用医生已告知的受限制药物、食物等；告诉研究医生自己最近是否参与其他研究，或目前正参与其他研究。

As a participant, it is your responsibility to provide accurate information about your medical history and current physical condition. You should inform the research doctor of any discomfort you experience during the study. You should not take any restricted medications or foods that the doctor has informed you about. Inform the research doctor if you have recently participated in other studies or are currently participating in other research.

八、相关联系方式
Section Eight: Contact Information

如果您有与本研究相关的任何问题，请通过电话 15710668779 与 闫丽娟医师 联系。

如果您有与自身权利/权益相关的任何问题，或者您想反映参与本研究过程

中遭遇的困难、不满和忧虑，或者想提供与本研究有关的意见和建议，请联系伦理委员会，联系电话： 0592-2137569，电子邮件： xdfyec@sina.com。

If you have any questions related to this study, please contact Dr. Lijuan Yan at the phone number 15710668779. If you have any concerns about your rights or interests, or if you wish to report difficulties, dissatisfaction, or anxieties encountered during your participation in this study, or if you wish to provide feedback or suggestions related to this study, please contact the Ethics Committee at 0592-2137569 or xdfyec@sina.com.

知情同意书·同意签字页

Informed Consent Form · Consent Signature Page

我已被告知此项研究的目的、背景、过程、风险及获益等情况。我有足够的时间和机会进行提问，问题的答复我很满意。

I have been informed about the purpose, background, procedures, risks, and benefits of this study. I have had sufficient time and opportunity to ask questions, and I am satisfied with the answers provided.

我也被告知，当我有问题、想反映困难、顾虑、对研究的建议，或想进一步获得信息，或为研究提供帮助时，应当与谁联系。

I have also been informed that if I have questions, wish to report difficulties, express concerns, provide suggestions for the study, seek further information, or offer assistance to the research, I should contact the designated person.

我已经阅读这份知情同意书，并且同意参加本研究。

I have read this informed consent form and agree to participate in this study.

我知道我可以选择不参加此项研究，或在研究期间的任何时候无需任何理由退出本研究。

I am aware that I can choose not to participate in this study or withdraw from it at any time during the study without providing any reason.

我已知道如果我的状况更差了，或者我出现严重的不良反应，或者我的研究医生觉得继续参加研究不符合我的最佳利益，他/她会决定让我退出研究。无需征得我的同意，资助方或者监管机构也可能在研究期间终止研究。如果发生该情况，医生将及时通知我，研究医生也会与我讨论我的其他选择。

I understand that if my condition worsens, or if I experience severe adverse reactions, or if my research doctor deems that my continued participation is not in my best interest, he/she may decide to withdraw me from the study. The sponsor or regulatory authorities may also terminate the study during its course without obtaining my consent. In such a case, I will be promptly informed by the doctor, and my other options will be discussed with me.

我将得到这份知情同意书的副本，上面包含我和研究者的签名。

I will receive a copy of this informed consent form, containing both my signature and that of the researcher.

受试者签名：____________________　　　　日期：____________

Participant's Signature: ________________　　　　Date:______________

联系电话：________________
Contact Phone:__________________

（注：如果受试者无行为能力或限制行为能力时，则需监护人在以下监护人签字处签名）

(Note: If the participant lacks legal capacity or has limited legal capacity, the legal guardian's signature is required below.)

监护人签字：_________________ 日期：____________
Guardian's Signature: ________________ Date: _______________

联系电话：____________________
Contact Phone: _________________

我已准确地将这份文件告知受试者，他/她准确地阅读了这份知情同意书，并证明该受试者有机会提出问题，他/她是自愿同意的。

I have accurately explained this document to the participant, and he/she has read it accurately and confirmed that the participant had the opportunity to ask questions and that he/she consented voluntarily.

研究者签名：__________________ 日期:_______________
Researcher's Signature:_____________ Date:________________

联系电话：__________________
Contact Phone:________________
